# Reoperations for Long-Term Complications Following Laparoscopic Adjustable Gastric Banding: Analysis of Incidence and Causality

**DOI:** 10.7759/cureus.8127

**Published:** 2020-05-14

**Authors:** Logan T Mellert, Maureen Cheung, Lindsay Berbiglia, Ashley Shoemaker, Deborah Douglas, Mark Pozsgay, John Zografakis, Adrian Dan

**Affiliations:** 1 Bariatric and Minimally Invasive Surgery, Summa Health, Akron, USA; 2 Cardiothoracic Surgery, The Ohio State University Wexner Medical Center, Columbus, USA; 3 General Surgery, Lee Health, Cape Coral, USA; 4 Bariatric Surgery, Summa Health, Akron, USA

**Keywords:** lagb, band malfunction, long-term maintenance, band reintervention, comparison to primary procedure, band conversion, sleeve gastrectomy, roux-en-y gastric bypass, laparoscopic adjustable gastric banding

## Abstract

Background: Laparoscopic adjustable gastric banding (LAGB) gained popularity in the early 2000s as a purely restrictive procedure with modest weight loss. The potential for complications requiring reoperation has since become evident. A retrospective review was performed to determine the incidence of long-term complications and predictive factors requiring surgical reintervention after LAGB.

Methods: Institutional review board approval was obtained, and a retrospective review of 200 consecutive patients undergoing LAGB over a period of six years was conducted at a single institution with American Society of Metabolic and Bariatric Surgery Center of Excellence designation. Data were collected on patient characteristics, comorbid conditions and complications requiring reintervention. Statistical analysis was performed using SPSS Statistics software (IBM Corp., Armonk, NY).

Results: Of the 200 patients, 176 (90.7%) were female with an average age of 53.6 years and preoperative body mass index (BMI) of 44.2 kg/m^2^. The average follow-up was 46 months. Complications occurred in 55 (28.4%) patients with band slippage/prolapse as the most common need for reoperation. Younger age, lack of comorbidities and diet/exercise compliance were associated with reintervention.

Conclusions: LAGB has a high rate of reoperation secondary to complications associated with younger age. Alternative bariatric procedures may be more appropriate in these patients who have fewer comorbid conditions and are motivated to improve his or her health.

## Introduction

The disease of obesity and its related comorbid conditions represent the most significant public health threat of our time [1]. Weight loss surgery has been proven to be the most effective and consistent treatment for patients seeking long-term weight loss and has demonstrated a significant impact on patient longevity [2,3]. Currently, several types of procedures are employed that vary in the degree of restriction and malabsorption [4]. The laparoscopic adjustable gastric band (LAGB) obtained FDA approval in the United States in 2001. Over the past 18 years, it has been utilized as a purely restrictive procedure for the treatment of obesity with reasonable weight loss results [5-7]. With more indwelling LAGBs, the potential for long-term complications requiring surgical intervention has become evident [8-12]. Predictive factors of these complications and reoperative rates have not fully been determined in the American patient population. The specific aims of this study are to provide clinicians and patients with incidence rates of long-term complications and predictive factors requiring surgical reintervention after LAGB.

## Materials and methods

Institutional review board (IRB) approval to conduct the study was obtained at a university affiliated tertiary care center with an American Society of Metabolic and Bariatric Surgery (ASMBS) Center of Excellence designation and subsequent Metabolic and Bariatric Surgery Accreditation and Quality Improvement Program (MBSAQIP) accreditation. A retrospective chart review was performed on 200 consecutive patients undergoing LAGB from 2005 to 2011. Data were collected through March 2015. Patients were considered lost to follow-up if no data were available at the one-year appointment. Data included age, gender, preoperative body mass index (BMI), excess body weight (EBW), % excess body weight lost (%EWL) and preoperative comorbidities: diabetes mellitus (DM), hypertension (HTN), obstructive sleep apnea (OSA), hyperlipidemia (HLD) and gastroesophageal reflux disease (GERD). The primary endpoint was need for surgical intervention due to a complication from LAGB. Complications resulting in reintervention were recorded and classified as band slippage/prolapse, weight loss failure, band intolerance, port/tube complications and gastric erosion. Reinterventions were further analyzed for conversion to an alternative bariatric procedure (laparoscopic Roux-en-Y gastric bypass [LRYGB] or laparoscopic sleeve gastrectomy [LSG]). Compliance with diet and exercise programs was also reported.

Data were tabulated in Excel, and analyses were performed using the SPSS Statistics software (IBM Corp., Armonk, NY). Pearson’s chi-squared test for association was used to calculate p-values if expected cell frequency was greater than or equal 5. If any expected cell frequency was greater than 5, Fisher’s exact method was employed. The Anderson-Darling statistic compared several distributions (exponential, normal and three-parameter Weibull) to determine fit. P-values from the Anderson-Darling Normality test determined if two-independent sample t-test or Mann-Whitney’s test was employed. A p-value of <0.05 was defined as statistically significant.

## Results

A total of 200 patients were studied with a mean follow-up of 46 months (range = 2-109) (Table 1). Of all participants, six (3%) patients did not have 12-month data available and were considered lost to follow-up and excluded from the study. Noted in the series were three deaths, none of which occurred within the 12-month initial operative period and none were associated with band complications.

**Table 1:** Descriptive Statistics of Patients Undergoing LAGB BMI, body mass index; EBW, excess body weight, LAGB, laparoscopic adjustable gastric banding; Std., standard deviation

Variable	Mean (Std.)	Median	Range
Months of follow-up	46.53 (±26.84)	44	(2, 109)
Age	53.60 (±11.61)	54	(24, 77)
Preoperative height (in.)	64.20 (±3.00)	64	(57.5, 75)
Preoperative weight (lbs)	259.33 (±40.35)	252.3	(184, 377.8)
Preoperative BMI	44.187 (±5.57)	43.05	(35.1, 62.1)
Preoperative EBW (lbs)	127.16 (±35.56)	121.1	(65, 232)
Weight at 12 months (lbs)	210.6 (±36.52)	205	(137, 340)
BMI at 12 months	35.876 (±5.35)	35.2	(24.8, 57.5)
EBW at 12 months (lbs)	78.78 (±32.19)	74.5	(13, 206)

Of all participants, 176 patients (90.7%) were female with a mean age of 53.6 years (standard deviation [SD] = 11.6, range = 24-77) and the mean preoperative BMI was 44.2 kg/m^2^ (SD = 5.6, range = 35.1-62.1). Patients with preoperative comorbidities (Table 2) included 129 (66.5%) HTN, 94 (48.5%) OSA, 92 (47.4%) GERD and 67 (34.5%) DM. LAGB complications requiring reoperation (Table 3) occurred in 55 (28.4%) patients. Reasons for reintervention included 24 (12.4%) band slippage/prolapses, 19 (9.8%) weight loss failures, five (2.6%) band intolerances, five (2.6%) port/tubing complications and two (1.0%) band erosions.

**Table 2:** Preoperative Sex, Comorbidities, Compliance and Need for Reintervention GERD, gastroesophageal reflux disease; OSA, obstructive sleep apnea

Variable		No. of Patients	Percentage
Sex			
	Male	18	9.3%
	Female	176	90.7%
Diabetes			
	Yes	67	34.5%
	No	127	65.5%
Hypertension			
	Yes	129	66.5%
	No	65	33.5%
OSA			
	Yes	94	48.5%
	No	100	51.5%
Hyperlipidemia			
	Yes	85	43.8%
	No	109	56.2%
GERD			
	Yes	92	47.4%
	No	102	52.6%
Exercise compliance			
	Yes	90	46.4%
	No	104	53.6%
Diet compliance			
	Yes	96	49.5%
	No	98	50.5%
Reintervention			
	Yes	55	28.4%
	No	139	71.6%

 

**Table 3:** Complications Requiring Reintervention

Complication	n	Percentage
Band slippage/prolapse	24	12.37%
Weight loss failure	19	9.79%
Band intolerance	5	2.58%
Port/tubing complication	5	2.58%
Band erosion	2	1.03%
Total reintervention	55	28.35%

Patients who required reintervention (Table 4) had a greater preoperative weight (268.55 lbs vs. 255.69, p < 0.016), but there was no significant difference in BMI (44.01 vs. 44.64, p = 0.681). These patients were younger (48.20 vs. 55.73, p < 0.001), had higher 12-month %EWL (44.58% vs. 36.62%, p = 0.002) and were more compliant with diet (Table 5, p = 0.031) and exercise (p = 0.038) than the no intervention group. There was a trend towards lower 12-month BMI in the reintervention group (p = 0.065); however, this did not reach statistical significance. Patients requiring reintervention were less likely to have HTN (49.1% vs. 73.4%, p = 0.001), DM (23.6% vs. 38.8%, p = 0.045) and HLD (30.9% vs. 48.2%, p = 0.05) compared with the no reintervention group. There was no statistically significant difference in sex, preoperative OSA or GERD between the groups.

**Table 4:** Descriptive Statistics Between LAGB Patient’s Not Requiring and Requiring Reintervention AD, Anderson-Darling; BMI, body mass index; EBW, excess body weight; LABG, laparoscopic adjustable gastric banding; Std., standard deviation

	Mean ± Std.			Median			P-Value	
Variable	No Reintervention (n = 139)	Reintervention (n = 55)		No Reintervention (n = 139)	Reintervention (n = 55)		AD Normality Test	Mann-Whitney's Test
Age	55.734 ± 10.76	48.20 ± 12.01		56	47		<0.005	0.000
Preoperative weight (lbs)	255.69 ± 41.11	268.55 ± 36.16		250.8	262.0		<0.005	0.016
Preoperative BMI	44.01 ± 5.49	44.64 ± 5.78		43.3	42.9		<0.005	0.681
Preoperative EBW (lbs)	124.85 ± 36.24	132.97 ± 33.38		118.0	127.4		<0.005	0.070
Weight at 12 months (lbs)	211.33 ± 38.46	208.77 ± 31.34		205	205		<0.005	0.911
BMI at 12 months (lbs)	36.35 ± 5.55	34.69 ± 4.62		35.7	34.1		<0.005	0.065
EBW at 12 months (lbs)	80.64 ± 34.05	74.07 ± 26.64		75	68		<0.005	0.252

**Table 5:** Relative Risk, Risk Difference and Attributable Proportion of Reintervention by Sex, Comorbidities, Exercise and Diet Compliance CI, confidence interval; GERD, gastroesophageal reflux disease; OSA, obstructive sleep apnea *Preventive fraction

Variable		No Reintervention (n = 139)	Reintervention (n = 55)	Relative Risk (95% CI)	P-Value (Chi-Squared)	Risk Difference	Attributable Proportion
Sex							
	Male	11	7	1.43 (0.76-2.67)	0.298	11.62%	29.87%
	Female	128	48	1.00			
Diabetes							
	Yes	54	13	0.59 (0.34-1.01)	0.045	-13.67%	41.33%*
	No	85	42	1.00			
Hypertension							
	Yes	102	27	0.49 (0.31-0.75)	0.001	-22.15%	51.41%*
	No	37	28	1.00			
Hyperlipidemia							
	Yes	67	17	0.62 (0.38-1.01)	0.050	-12.77%	37.62%*
	No	72	37	1.00			
OSA							
	Yes	68	26	0.95 (0.61-1.49)	0.836	-1.34%	4.62%*
	No	71	29	1.00			
GERD							
	Yes	63	29	1.24 (0.79-1.94)	0.352	6.03%	19.13%
	No	76	26	1.00			
Exercise							
	Yes	58	32	1.61 (1.02-2.54)	0.038	13.44	37.80%
	No	81	23	1.00			
Diet							
	Yes	62	34	1.65 (1.04-2.63)	0.031	13.99	39.50%
	No	77	21	1.00			

For the 55 patients undergoing reintervention, 25 (46%) underwent an alternative bariatric procedure (Table 6). These patients tended to have a lower preoperative weight (117.7 vs. 125.71, p = 0.083). There was no statistical significance between preoperative BMI and 12-month %EWL. There was no statistical difference in comorbidities, sex or exercise/diet compliance between these groups (Table 7). The average time to conversion to an alternative bariatric procedure was 55.5 (range = 25.8-87.5) months. The majority of these patients (21) underwent conversion to a LRYGB, while only four patients had an LSG.

**Table 6:** Descriptive Statistics of Reintervention Patients Undergoing Alternative Bariatric Procedures AD, Anderson-Darling; BMI, body mass index; EBW, excess body weight; Std., standard deviation *Two-independent sample t-test

	Mean ± Std.			Median			P-Value	
Variable	No Bariatric Procedure (n = 30)	Bariatric Procedure (n = 25)		No Bariatric Procedure (n = 30)	Bariatric Procedure (n = 25)		AD Normality Test	Mann-Whitney's Test
Age	49.33 ± 11.36	46.84 ± 12.85		48.5	47.0		0.597	0.454*
Preoperative weight (lbs)	125.71 ± 16.62	117.70 ± 15.41		125.68	116.82		0.030	0.083
Preoperative BMI	45.95 ± 6.76	43.06 ± 3.87		43.7	42.8		<0.005	0.265
Preoperative EBW (lbs)	64.37 ± 16.08	55.73 ± 12.78		62.95	55.73		0.017	0.061
Weight at 12 months (lbs)	94.98 ± 13.75	94.80 ± 15.11		92.50	97.73		0.071	0.965*
BMI at 12 months	34.70 ± 5.18	34.67 ± 3.96		33.55	34.20		0.100	0.982*
EBW at 12 months (lbs)	33.78 ± 12.76	33.54 ± 11.55		29.00	32.73		0.128	0.943*

**Table 7:** Relative Risk, Risk Difference and Attributable Proportion of Bariatric Surgery by Sex, Comorbidities, Exercise and Diet CI, confidence interval; GERD, gastroesophageal reflux disease; OSA, obstructive sleep apnea *Preventive fraction †Fisher’s exact method

Variable		No Bariatric Surgery (n = 30)	Bariatric Surgery (n = 25)	Relative Risk (95%CI)	P-Value (Chi-Squared)	Risk Difference	Attributable Proportion
Sex							
	Male	2	5	0.86 (0.69-1.07)	0.226†	-13.33%	14.29%*
	Female	28	20	1.00			
Diabetes							
	Yes	7	6	1.03 (0.40-2.67)	0.954	0.67%	2.78%
	No	23	19	1.00			
Hypertension							
	Yes	16	11	0.83 (0.47-1.44)	0.491	-9.33%	17.5%*
	No	14	14	1.00			
Hyperlipidemia							
	Yes	10	8	0.96 (0.45-2.06)	0.916	-1.33%	4%*
	No	20	17	1.00			
OSA							
	Yes	14	12	1.02 (0.59-1.80)	0.921	1.33%	2.78%
	No	16	13	1.00			
GERD							
	Yes	17	12	0.85 (0.51-1.42)	0.521	-8.67%	15.3%*
	No	13	13	1.00			
Exercise							
	Yes	19	13	0.82 (0.51-1.31)	0.369	-11.33	18.9%*
	No	11	12	1.00			
Diet							
	Yes	20	14	0.84 (0.55-1.29)	0.418	-10.67	16%*
	No	10	11	1.00			

## Discussion

LAGB is known to provide moderate weight loss with an excellent perioperative safety profile [5-7]. Early studies revealed short-term superiority with the LAGB in regards to weight reduction (43-78 %EWL at three years), remission of diabetes and other obesity-related comorbidities compared with medical therapy alone [13-15]. Perioperative mortality was found to be <0.05% in these studies [15]. Despite this, as more data became available, LABG showed poor long-term efficacy. Ten-year data from Froylich et al. revealed no improvement in comorbidities and worsening of GERD-related symptoms; 59.4% of the patients had their band removed, and 30% of those went on to have an alternative bariatric procedure [16]. With other studies reporting high rates of reintervention between 19.2% and 33.1%, bariatric procedures with more durable weight loss outcomes were advocated [8,9,17].

LSG and LRYGB were studied in comparison to the LAGB and found to be superior. LSG and LRYGB showed greater weight loss with improvement or remission of comorbidities compared with LAGB [18-21]. Tice et al. showed superior %EWL (76% vs. 48%) and diabetes resolution (78% vs. 50%) comparing LRYGB and LAGB. Although perioperative complications were higher for LRYGB (9% vs. 5%), LRYGB was associated with fewer long-term reoperations (16% vs. 25%) and higher patient satisfaction [19]. LRYGB achieved greater weight loss and BMI reduction five years after surgery compared with LAGB [20]. In the super obese population (BMI > 50), there was no difference in early complications between LRYGB and LAGB; however, LRYGB was again associated with higher EWL at 12 months (54.7% vs. 31.5%) [21].

For patients requiring reintervention and seeking further weight loss after LAGB failure, conversion to LSG or LRYGB in a one- or two-stage procedure is feasible [22-25]. Several studies have shown that conversion to another bariatric procedure is safe with low morbidity (1.1%) and mortality (<1%) [24,25]. Both conversion procedures have similar complication rates, hospital stay and early weight loss [22-25]. Additionally, the EWL in converted patients at two years was similar, approximately 60% [22,25].

In our study, long-term complications from LAGB leading to surgical reintervention occurred with significant frequency, with more than 28.4% of the patients requiring surgery within a 46-month average follow-up. The most common reason for reintervention was band slippage or prolapse followed closely by weight loss failures. Less common were band intolerances, port/tubing complications and band erosions. Causes for reintervention appear consistent with reported literature; however, direct comparison is hampered by variability in reporting of complications in these studies [8,9,26]. Of the patients who experienced weight loss failure, 84% (n = 16) underwent an alternative bariatric procedure compared with 33% (n = 8) of band slippage/prolapse patients. Only one patient with band intolerance underwent an alternative procedure for severe GERD associated with the LAGB. Of those undergoing an alternative bariatric procedure, 84% (n = 21) elected for LRYGB compared with the LSG (n = 4). While difficult to quantify, there appeared to be a bias towards conversion to LRYGB due to concern of trading one restrictive procedure (LAGB) for another (LSG). Given equivocal two-year %EWL, which is evident in newer literature, this is likely an unfounded concern [24,25].

Overall, patients in this study experiencing band complications requiring reintervention were younger, had a higher preoperative weight and less comorbidities (lower rates of DM, HTN and HLD). At 12 months, these patients tended to have a lower %EWL and were statistically significantly more compliant with prescribed diet and exercise routines. Of this reintervention group, those with a lower %EWL at 12 months were more likely to undergo a conversion to an alternative bariatric procedure. These data suggest that in younger, healthier patients who are motivated about weight loss, LAGB may not be the ideal initial procedure as it is associated with higher reintervention rates over time. As 45.5% of this population underwent an alternative bariatric procedure, an LRYGB or LSG may be more satisfactory to meet these patients’ long-term weight loss goals.

The limitations of our study include its retrospective nature without randomization or a control group. Some patients were lost to follow-up and may have presented to outside institutions for complication care and therefore could not be accounted. Additionally, no data regarding quality of life or weight loss satisfaction were collected. These factors may also influence a patient’s decision when choosing a bariatric procedure.

## Conclusions

Although LAGB is not as common as it was a short time ago, the procedure is still performed today and most practicing bariatric surgeons will encounter patients with indwelling bands. Clinicians caring for these patients should be aware of the complications necessitating reoperation and the patient characteristics associated with these complications. LAGB is associated with high long-term complication rates, and our study suggests that alternative bariatric procedures may be more appropriate in younger patients with fewer comorbid conditions who are motivated to lose weight. While LAGB may still have a limited role in a select subset of patients, given the high frequency of potential complications, it should not be presented as the only option within a bariatric program. Our findings may guide clinicians and patients in utilizing alternative bariatric and metabolic procedures which have been shown to have superior efficacy with much lower rates of surgical reintervention.
